# Notes from Australia

**Published:** 1898-09-17

**Authors:** 


### THE INSTITUTIONAL WORKSHOP.

### NOTES FROM AUSTRALIA.

### MELBOURNE.

The health of the colony since the beginning of the present year has been indifferent. Such an epidemic of measles has never been known before—lasting quite into the winter, and only now beginning to abate. The disease has been exceedingly virulent, with a large percentage of deaths, and has attacked people for the second, and occasionally for the third time, adult victims being nearly as numerous as children. Diphtheria was also plentiful, and typhoid, which recurs with the greatest regularity every summer, was prolonged well into winter. In many country places the State school had to be closed for four, six, or eight weeks. The diseases have been quite as severe in the country as in the metropolis, where the system of water sewerage is now being rapidly completed. This is not wonderful to anyone acquainted with the country districts of Victoria, where the cesspit is in all but universal use, where there is no system for the disposal of refuse, and water is frequently used from stagnant ponds, and all insanitary conditions are aggravated by the heat of the climate. The wide spaces of the country alone save it from greater disaster.

The Queen Victoria Hospital for Women will shortly start an in-patients' department. The trustees of the £3,000 collected last year have expended £2,000 in purchasing the building formerly used as the Governesses' Institute. This hospital, which has had an out-patients' department for two years past, is entirely officered by medical women.

The present season is one of annual general meetings. Most of the medical and benevolent charities are in debt, and grievously disappointed at the smallness of the sums received from Government this year, whilst the Premier is at his wit's end trying to content all the committees. The Melbourne General Hospital, the largest and eldest of these institutions amongst us, is £20,000 in debt. The average cost per bed per annum is £75 12s. 9d., the highest in Victoria. The committee of the Children's Hospital find it necessary to appeal for funds to carry out the additions to the building so urgently needed.

A youth of 20 died in the Melbourne Eye and Ear Hospital on July 21st immediately after an operation for the removal of an eye. Chloroform was used. Deaths under similar circumstances are no more unusual here than in Great Britain, and it was probably only the outcry of the boy's mother that she had not given permission for the operation that drew attention to this case more than to many others that have happened. The inquest elicited the fact that the heart and other organs were perfectly healthy; that the chloroform was administered by means of a flannel mask "in the usual way," less than usual being given; and that death, according to the doctor who performed the autopsy, resulted from asphyxia syncope, "the most frequent cause of death during the administration of chloroform." Artificial respiration had been kept up for 35 minutes. A man died in Adelaide on July 23rd under nearly identical circumstances, having never regained consciousness after chloroforming for the reduction of a dislocated shoulder. A newspaper correspondence was started in one paper by "Inquirer," and in another by "Surgeon," about the youth's case; but few replies or opinions were elicited other than those of the chloroformist and the operator, who stated that they had done all that could be done. Evidently an inquiry into the frequency of death under chloroform, such as The Hospital has instituted, is required elsewhere than in England.

The Benevolent Asylum—to which old or infirm people are sent—really the equivalent of the British workhouse, has had a thorough thrashing out of its affairs. With one exception its elections of committee have gone uncontested for over 20 years, and the state of affairs, with regard to admission of inmates, tenders for provisions and such, had become a public scandal. The average number of inmates is 700, and the annual expenditure £10,000. The first indication of public opinion was a proposition that four women should be elected on the committee of twelve. This idea was scornfully rejected by the sitting committee. The Charity Organisation Society took the matter up, and issued a "ticket." There were seven vacancies, and 19 candidates came forward. The elections took place yesterday, August 9th, with the result that the Charity Organisation Society's "ticket" was adopted in its entirety. The ladies elected were Mrs. Bevan, wife of the Rev. Dr. Bevan, of the Congregational Church; Dr. Constance Stone, the first woman doctor to practise in Melbourne; and Dr. Lilian Alexander, one of Melbourne's own medical *alumni* Messrs. John Barwise, Isaac Barnet, and C. F. Heales, former committeemen, who for years demanded full and open inquiry, and thereby lost their seats, and Mr. Martin Hood, who retired from the committee only a year ago, and has always been a strenuous advocate for improvement in the mode of obtaining provisions were the other four. Amongst the dozen rejected were Mr. Theyre á Beckett Weigall, formerly Curator of Intestate Estates.

A European leper has been discovered in Brisbane, Queensland, and sent to the station on Stradbroke Island. He is a married man of 33, with six children. European lepers are not unknown, especially in the northern parts of Australia, but happily they are rare.

Dr. T. H. Hutchison, who was considerably over 80 years of age, died August 8th in the public hospital at Kyneton, a small country town about 60 miles from Melbourne. Dr. Hutchison came to Australia in the forties, settled in the Trentham District about the time of the first gold rushes, and practised his profession until about 10 years ago, when he became totally blind.

